# The voltage-gated sodium channel, para, limits *Anopheles coluzzii* vector competence in a microbiota dependent manner

**DOI:** 10.1038/s41598-023-40432-x

**Published:** 2023-09-04

**Authors:** Luisa Nardini, Emma Brito-Fravallo, Pascal Campagne, Adrien Pain, Corinne Genève, Kenneth D. Vernick, Christian Mitri

**Affiliations:** 1 Genetics and Genomics of Insect Vectors Unit, Department of Parasites and Insect Vectors, CNRS, Institut Pasteur, UMR2000, Université de Paris, 75015 Paris, France; 2 Center of Bioinformatics, Biostatistics and Integrative Biology, Institut Pasteur, Université de Paris, 75015 Paris, France

**Keywords:** Malaria, Parasite host response, RNAi, Metagenomics, Transcriptomics

## Abstract

The voltage-gated sodium channel, para, is a target of DDT and pyrethroid class insecticides. Single nucleotide mutations in *para*, called knockdown resistant or kdr, which contribute to resistance against DDT and pyrethroid insecticides, have been correlated with increased susceptibility of *Anopheles* to the human malaria parasite *Plasmodium falciparum*. However, a direct role of *para* activity on *Plasmodium* infection has not yet been established. Here, using RNA-mediated silencing, we provide in vivo direct evidence for the requirement of wild-type (wt) para function for insecticide activity of deltamethrin. Depletion of wt para, which is susceptible to insecticide, causes deltamethrin tolerance, indicating that insecticide-resistant kdr alleles are likely phenocopies of loss of para function. We then show that normal para activity in *An. coluzzii* limits *Plasmodium* infection prevalence for both *P. falciparum* and *P. berghei*. A transcriptomic analysis revealed that para activity does not modulate the expression of immune genes. However, loss of para function led to enteric dysbiosis with a significant increase in the total bacterial abundance, and we show that para function limiting *Plasmodium* infection is microbiota dependent. In the context of the bidirectional “enteric microbiota-brain” axis studied in mammals, these results pave the way for studying whether the activity of the nervous system could control *Anopheles* vector competence.

## Introduction

Voltage-gated sodium channels (VGSC) are transmembrane proteins mediating electrical potential (voltage) in excitable cells such as neurons and muscle cells and participate in activities such as muscle contraction^1–3^, cell proliferation^4^ and cognition^5,6^. Pyrethroids represent one of the most useful insecticide classes and have been used intensively in agricultural and public health settings^7–9^. Deltamethrin is a pyrethroid that binds to the VGSC, preventing its transition from an activated to an inactivated state, leading to persistent depolarized cell membranes that paralyze and kill the insects^10,11^.

Malaria is a disease caused by *Plasmodium* parasites, which are transmitted to humans by the bite of infectious *Anopheles* mosquito females and has killed a predicted 619,000 persons in 2021^12^. The use of indoor residual spraying and long-lasting insecticide-treated nets have been the foundation of mosquito vector control interventions and have provided major gains in reducing malaria transmission. However, this gain has been hampered by the development and spread of insecticide resistance in mosquito vector populations^12^. *Anopheles* resistance to the pyrethroid insecticides is conferred by metabolic detoxification through the action of the P450 monooxygenases or glutathione-S-transferases (GSTs)^13–17^, by point mutations of the VGSC that inhibit pyrethroid binding at the target site^18,19^ and by cuticular resistance^20^. This VGSC is also called para due to the paralytic phenotype observed in *Drosophila* VGSC mutants^21,22^. Several mutations identified in the VGSC lead to pyrethroid resistance known as knock-down resistance (kdr) alleles: the L1014F and L1014S – a leucine to phenylalanine substitution^19^ and a leucine to serine substitution^18^. More recently, a novel mutation I1527T, adjacent to a predicted pyrethroid-binding site, has been found *An. coluzzii* from Ghana, Burkina Faso, Cote d'Ivoire and Guinea^23^. An additional kdr mutation, formerly known as N1575Y, the so called “super kdr”, occurs with and intensifies the effect of the L995F-mediated pyrethroid resistance in *An. gambiae* from southern Cote d’Ivoire^10,24,25^.

The relationship between the insecticide resistance phenotype and *Anopheles* vector competence to *Plasmodium falciparum* infection is not well understood. In laboratory populations of *An. gambiae*, the presence of *kdr* has been correlated with increased infection susceptibility of *Plasmodium*^26,27^. In wild *An. gambiae *sensu lato mosquitoes, the *kdr* resistance allele was associated with greater susceptibility to *Plasmodium*^28,29^, while in another study no relationship was found^30^. Despite the genetic association, a functional link between activity of para and *Anopheles* vector competence for *Plasmodium* has not been shown. We previously performed functional genomics studies of the para gene where we did not detect a phenotype with *P. falciparum* infection^31^. As a transmembrane protein, the half-life of the VGSC is known to be long^32,33^, and lacking a specific antibody against the para protein, one limitation of the study in^31^ was that we were not able to measure para protein levels after para gene silencing. In addition, a new annotation of the para gene predicted additional splice variants and new transcripts that were not targeted by the silencing procedure used in the 2015 study.

Here, to clarify the relationship between *para* activity and *Plasmodium* infection in *Anopheles* mosquitoes, we designed a silencing strategy that targeted all of the annotated transcripts. We showed that disruption of para activity by RNAi increased *Anopheles* vector competence for infection for both *P. falciparum* and *P. berghei*. We also showed that para silencing led to dysbiosis of the enteric bacterial microbiome and that para activity in limiting *Plasmodium* infection is microbiota dependent. To our knowledge, this is the first functional study linking para activity with *Anopheles* vector competence for *Plasmodium*.

## Methods

### Ethics Statement/ethical considerations

In conducting these experiments, we adhered to the Guide for the Care and Use of Laboratory Animals of the European Union and the French Government. The methods and experiments were approved by the French Ministry of Higher Education, Research and Innovation under the agreement reference (APAFIS #30954-2021040810159180 v1). The study is reported in accordance with ARRIVE guidelines (https://arriveguidelines.org) ^34^.

### Mosquitoes

The Ngousso strain (*An. coluzzii*) was colonized in 2006 from mosquitoes collected in Yaoundé, Cameroon. The mosquitoes are insecticide susceptible, and the colony was reared under standard insectary conditions (26 °C, 12:12 L:D, and 70% relative humidity) by the Centre for the Production and infection of *Anopheles* (CEPIA) of the Institute Pasteur in Paris, France.

### Synthesis of double stranded RNA and Nanoinjection

The *para* gene (AGAP004707), located on chromosome arm 2L, is composed of 39 exons, which transcribe 13 different messenger RNAs (mRNA) through alternate splicing. To maximize the efficacy of the gene silencing, two double stranded (ds) RNA for *para* were designed and synthesized for targeting each two distinct regions along the *para* gene with the aim to recognize all 13 transcripts. Each of the dsPara and the dsGFP used as control were prepared using the MEGAscript™ RNAi Kit (Invitrogen by Thermo Fisher Scientific) as described in^35^. Primers bearing the T7 RNA polymerase recognition sequence were designed to amplify ~ 604 (Para1) and ~ 730 (Para2) base pairs regions. All the primer sequences used for the synthesis of the T7-matrix are listed in Table 1. For gene silencing procedure, one to three day old cold-anaesthetized mosquitoes were injected using the NanoJect II (Drummond) either with 1 μg of an equal quantity of the two dsPara (500 ng each per mosquito) or with 1 μg of dsGFP for the control group. dsPara1 and dsPara2 each at 10 μg/μL were used to perform the mix of dsPara used to inject the mosquitoes and 100 nL of this dsRNAmix were injected in each mosquito female. The mosquitoes were injected into the thorax and maintained on sugar water until required for downstream experiments. Efficiency of silencing was determined from cDNA by RT-qPCR, using Total RNA extracted from mosquitoes, 4 days post-injection as described previously in^31^.

**Table 1:** Primers required for synthesis of dsRNA and detection of gene silencing by PCR.

Primer ID	Sequence	Citation
dsPara 1_Fwd	5′ TAA TAC GAC TCA CTA TAG G AG GGC TAT CCG GGA AAT TGT GG 3′	Mitri et al. ^31^
dsPara 1_Rev	5′ TAA TAC GAC TCA CTA TAG G TG AAG CG TCT GTT CCG CCT CC 3′	Mitri et al. ^31^
dsPara 2_Fwd	5′ TAA TAC GAC TCA CTA TAG GGA GA C CAC GTG TGT GGG ACT GTT GTT GG 3′	N/A
dsPara 2_Rev	5′ TAA TAC GAC TCA CTA TAG GGA GA C CAC CAG TGG CCA AGA AAA ATG GT 3′	N/A
dsGFP_Fwd	5′ GAA TTG TAA TAC GAC TCA CTA TAG GGC ATG GTG AGC AAG GGC GAG 3′	Mitri et al. ^35^
dsGFP_Rev	5′ GAA TTG TAA TAC GAC TCA CTA TAG GGC TTA CTT GTA CAG CTC GTC 3′	Mitri et al. ^35^
Silencing_Fwd	5′ CAC CAG ACA ATG ATA AGG GC 3′	N/A
Silencing_Rev	5′ CCT TCT TGA ACA TCT TCC GTA G 3′	N/A

Underlined sequences correspond to the T7 sequence required for the T7 RNA polymerase.

### The effect of para silencing on mosquito survival

In order to determine if silencing the *para* gene had any impact on mosquito survival, which might confound data from subsequent experiments, mosquitoes were injected with dsGFP or dsPara as described above, whereafter mosquitoes were maintained on sugar water (10%), ad libitum, for 14 days post injection. Three biological replicates were performed and for each replicate, mosquito mortality was monitored daily with in total 126 mosquitoes in dsGFP and 181 in dsPara. Kaplan–Meier survival curves were prepared and analysed in GraphPad Prism (Version 8.0.0 for Windows, graphpad.com) and the *p*-values of each replicate were combined using Fisher’s method (Fisher, 1925).

### World Health Organization Bioassays

In the absence of antibodies against the hydrophobic membrane channel, and in order to establish whether the gene silencing was effective at the protein level, we prepared modified WHO bioassays using deltamethrin (0.05%) obtained from University Sains Malaysia (Malaysia). The bioassays were carried out 4, 6 and 10 days post injection (~ 1 μg dsGFP or dsPara) and Ngousso mosquitoes were exposed for a period of 7 min, minimal exposure time required for the dsGFP control group to reach close to 100% mortality 24 h after deltamethrin exposure and limit binding saturation of the insecticide on the remaining para receptor in the dsPara background. Depending on mosquito availability and the number injected, between 10 and 25 mosquitoes were exposed per assay, and a minimum of 4 biological repeats were prepared. The mortality rate of the mosquitoes exposed at d-4, d-6 and d-10 was compared between the dsPara and dsGFP treated cohorts 24 h post-exposure, and the results were analysed using the Chi-Square test where *p*-values were empirically determined using 10^5^ Monte-Carlo permutations. Following independent statistical tests, the *p*-values from independent tests of significance were combined using the meta-analytical approach of Fisher^36^ when the direction of change of each independent replicate was concordant (e.g., for each independent replicate the dsPara displayed lower mortality rate than their paired dsGFP control ).

### *Plasmodium falciparum* gametocyte culture and mosquito infection

*P. falciparum* isolate NF54 was cultured using an automated tipper-table implemented in the CEPIA mosquito infection core facility of the Institut Pasteur, as previously described^31,35^. Briefly, fourteen days after initiating the subculture, gametocyte maturity was tested by exflagellation of microgametes, and parasitemia and numbers of mature male and female gametocytes were counted on Giemsa-stained slides. For experimental infection of mosquitos, 10 ml of medium containing mature gametocytes was centrifuged at 2000 rpm, and the cell pellet was resuspended in an equal volume of normal type AB human serum. The infected erythrocytes were added to fresh erythrocytes in AB human serum, mixed gently, and transferred to a membrane feeder warmed to 37 °C. At 4 days post-dsRNA injection, *An. coluzzii* mosquitoes were allowed to feed for 15 min, unfed females were discarded, and only fully engorged females were used for further analysis. Blood-fed mosquitoes were maintained at 26 °C and at 70% relative humidity on 10% sucrose solution.

### Rodent malaria infection with or without antibiotic treatments

One to three-days old female mosquitoes were injected with dsGFP or dsPara and 4 days post -injection *An. coluzzii* mosquitoes were fed on mice infected with *P. berghei* strain PbGFPCON^37^, which constitutively expresses green fluorescent protein (GFP). The mosquitoes were allowed to feed for 15 min. Unfed females were discarded, and only fully engorged females were maintained at 21 °C (*P. berghei*) and 70% relative humidity on 10% sucrose.

The same experiments were performed with mosquitoes treated with antibiotic. Briefly, immediately following adult emergence, mosquitoes were maintained on a 10% sucrose solution complemented with Penicillin 62.5 μg/mL, Streptomycin 100 μg/mL and gentamicin 50 μg/mL, and this solution was changed every day. The dsRNA injections and infectious feedings were performed as described above and only fully engorged females were maintained at 21 °C (*P. berghei*) and 70% relative humidity on 10% sucrose supplemented with the antibiotics.

### Analysis of phenotypes

Phenotypes were calculated from biological replicates of ≥ 30 dissected mosquitoes each and at least three independent replicates were performed for each species of *Plasmodium*. Mosquito midguts were dissected at 8 d post-infection. For *P. falciparum*, midguts were stained with 0.4% mercury dibromofluorescein (Sigma) and the number of oocysts was counted by light microscopy. For *P. berghei*, oocysts were counted by fluorescence microscopy. Phenotypes measured oocyst infection prevalence, which is the proportion of mosquitoes carrying ≥ 1 oocyst among the total number of dissected mosquitos, and oocyst intensity, which is the number of oocysts counted in mosquitoes with ≥ 1 oocyst. Differences in infection prevalence were statistically tested using the Chi-Square test, and analysis of oocyst intensity used the Wilcoxon signed rank non-parametric test. Statistical differences in prevalence and intensity were first tested independently for each independent replicate as described above and *p*-values were empirically determined using 10^5^ Monte-Carlo permutations. Following independent statistical tests, the *p*-values from independent tests of significance were combined using the meta-analytical approach of Fisher^36^ when the direction of change of each independent replicate was concordant (e.g., each independent replicate displayed higher infection prevalence than their paired GFP controls).

### Evaluation of total 16S and Enterobacteriaceae titers

Total 16S and Enterobacteriaceae 16S were measured in order to establish whether *para* transcript silencing had an impact and bacterial microbiome titers. Three-day old female mosquitoes were injected with dsPara or dsGFP, and after 4 days, the midguts were dissected and collected for DNA extraction. For midguts dissection, whole mosquitoes were submerged in 70% ethanol for 3 min, followed by 2 subsequent baths in sterile phosphate buffered saline. Intact (i.e. non-burst or damaged) midguts were collected in sterile Eppendorf tubes on dry ice, and midguts were retained. For each biological replicate, pools of ~ 18 midguts were extracted per treatment group (dsGFP and dsPara) and stored at -80 °C until processing. Three independent biological replicates were prepared. DNA was extracted using the DNeasy PowerSoil Kit (Qiagen). Universal primers (16S_V4q_F: 5′-GTG CCA GCM GCC GCG GTA A-3′ and 16S_V4q_R: 5′-GGA CTA CHV GGG TWT CTA AT-3′) for the V4 region of 16S rDNA were used in real-time qPCR reactions prepared as follows: 10 μl SYBR Green PCR Mix 2X (KAPA Sybr Fast 2X [Sigma-Aldrich]), 0.4 μl forward primer (10 μM), 0.4 μl reverse primer (10 μM), 8.2 μl nuclease-free H_2_O and 1ul template. Cycling conditions were: 95 °C/1 min followed by 40 cycles of [95 °C/3 s; 60 °C for 30 s; “plate-read”]. Enterobacteriaceae specific PCR was prepared in the same way but using the following primer pair—Entero16S_Fwd: 5′ CGT CGC AAG MMC AAA GAG 3′ and Entero16S_Rev: 5′ TTA CCG CGG CTG CTG GCA C 3′. All reactions were run in triplicate on the CFX Touch (Bio-Rad) and the relative expression was calculated by the 2-delta, delta Ct method after normalization against mosquito *ribosomal protein S7 (rpS7)*. Difference in delta Ct distribution across the independent biological replicates between (dsPara) and (dsGFP) samples was statistically tested using Student t-test.

### Transcriptional analysis

In order to investigate if there are pathways that might be influenced by loss of function in the voltage gated sodium channel, RNA-seq was carried out using material extracted from dsRNA-treated mosquitoes. Three-day old female females *Anopheles coluzzii* mosquitoes were injected with dsPara or dsGFP, and after 4 days, total RNAs from three biological replicates were extracted and sequenced. For each biological replicate, pools of ~ 18 mosquitoes were extracted per treatment group. RNA-seq was performed at the University of Minnesota Genomics Center (genomics.umn.edu) as previously described in^38^. Briefly, using Illumina’s Truseq RNA Sample Preparation Kit (Cat. # RS-122-2001), 1 µg of total RNA was oligo-dT purified using oligo-dT coated magnetic beads, fragmented and then reverse transcribed into cDNA. The cDNA was fragmented, blunt-ended, and ligated to indexed (barcoded) adaptors and amplified using 15 cycles of PCR. Final library size distribution was validated using capillary electrophoresis and quantified using fluorimetry (PicoGreen) and via q-PCR. Indexed libraries were then normalized, pooled and then size selected to 320 bp + /− 5% using Caliper’s XT instrument. Truseq libraries were hybridized to a paired end flow cell and individual fragments were clonally amplified by bridge amplification on the Illumina cBot. Once clustering was complete, the flow cell was loaded on the HiSeq 2000 and sequenced using Illumina’s SBS chemistry. Primary analysis of sequence reads and demultiplexing were done using CASAVA 1.8.2 and de-multiplexed FASTQ files were used for downstream analyses.

Raw read quality was assessed using FastQC version 0.11.5 (http://www.bioinformatics.babraham.ac.uk/projects/fastqc) and MultiQC version 0.7^39^. BWA-MEM version 0.7.7-r441 (https://arxiv.org/abs/1303.3997) was used for alignment against the reference genome of *Anopheles gambiae* str. PEST version AgamP4 (VectorBase), with default parameters. Genes were counted using featureCounts^40^ version 1.4.6-p3 with the annotation version AgamP4.7 and the parameters–t mRNA–g ID. Counts data were analyzed using R version 3.3.1 (R Core Team. R: A Language and Environment for Statistical Computing, R Foundation for Statistical Computing (2016)) and the Bioconductor package DESeq2 version 1.14.1^41^ with default settings. Genes with adjusted *p*-values below 0.05 were considered differentially expressed. For each pairwise comparison, raw *p*-values were adjusted for multiple testing using the Benjamini and Hochberg procedure^42^. Genes with adjusted *p*-values below 0.05 were considered differentially expressed.

## Results

### Loss of para function phenocopies insecticide allele resistance

**Figure 1:**
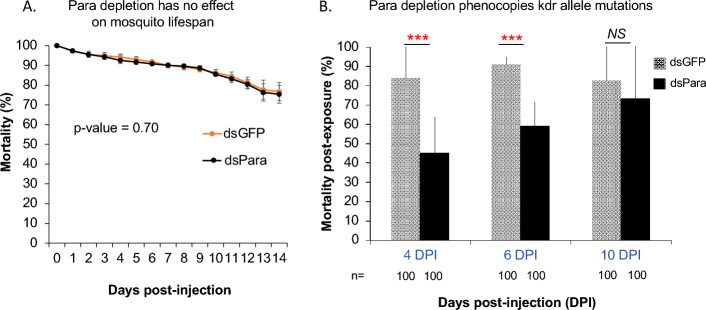
Depletion of para expression reduces deltamethrin sensitivity. (**A**) Daily mortality rates between dsGFP and dsPara mosquito groups were monitored for 14 days after dsRNA injection. No difference in mortality was found between dsGFP and dsPara. (**B**) Sensitivity to the pyrethroid deltamethrin (0.05%) was measured in dsGFP and dsPara by exposing the mosquitoes to the impregnated papers for 7 min, and 24h post-exposure, the mortality rate was counted in each group. The 7 min time exposure choice was made to avoid saturation of the para channels by the insecticide, and for which the control group is reaching close to 100% death. Mortality rate was significantly reduced in dsPara background at 4 and 6-days post-exposure, but not at 10 days.

1-day old female *An. coluzzii* Ngousso were injected with a mix of two dsRNA (dsPara) to ensure that all 13 alternative splices are targeted and for the control group, dsGFP were injected in mosquitoes. Silencing efficiency was first confirmed by RT-qPCR at 4 days post-injection (Fig. S1). We then measured and compared the survival between dsPara and dsGFP mosquito groups for 14 days post-injection. Mortality curves revealed the absence of significant fitness difference between dsPara and dsGFP treated mosquitoes (*p*-value = 0.7) (Fig. 1A).

To detect the effect of para depletion mediated by RNAi, and lacking protein-specific antibody, we tested mosquito sensitivity to the insecticide deltamethrin, known to target para receptors. In order to avoid saturation of the remaining receptor after para depletion, mosquitoes were exposed to deltamethrin (0.05%) for 7 min, which was the minimal exposure time that leads to almost 100% mortality in the dsGFP control group. Mosquitoes treated with dsPara showed significantly reduced mortality as compared to the dsGFP group, at d-4 (Chi2 = 46.86, DF = 8, *p* = 1.63 × 10^–07^; Table 2 and Fig. 1B) and d-6 (Chi2 = 33.50, DF = 8, *p* = 4.99 × 10^–05^; Table 2 and Fig. 1B) post-injection, but not at d-10 post-injection (Chi2 = 5.24, DF = 8, *p* = 0.73; Table 2 and Fig. 1B), suggesting that at this time point the transcript and protein abundance returned to normal levels (Fig. 1B). This reduction of deltamethrin sensitivity indicated that loss of para function can phenocopy kdr allele resistance.

**Table 2:** Statistical analysis of mortality phenotypes post-deltamethrin exposure following gene silencing.

Day post-injection	Conditions	Chi2 *p*-values of individual replicates	Combined *p*-value (Fisher method)
D-4	Rep1 dsGFP versus dsPara	Chi2 = 24.605, df = 1, *p*-value = 7.035E-07	Chi2 = 46.85, df = 8, ***p* = 1,63E-07**
	Rep2 dsGFP versus dsPara	Chi2 = 1.0218, df = 1, *p*-value = 0.3121	
	Rep3 dsGFP versus dsPara	Chi2 = 4.3283, df = 1, *p*-value = 0.03748	
	Rep4 dsGFP versus dsPara	Chi2 = 7.0086, df = 1, *p*-value = 0.008112	
D-6	Rep1 dsGFP versus dsPara	Chi2 = 10.859, df = 1, *p*-value = 0.0009832	Chi2 = 35.50, df = 8, ***p* = 4.99E-05**
	Rep2 dsGFP versus dsPara	Chi2 = 1.6606, df = 1, *p*-value = 0.1975	
	Rep3 dsGFP versus dsPara	Chi2 = 4.878, df = 1, *p*-value = 0.0272	
	Rep4 dsGFP versus dsPara	Chi2 = 6.6272, df = 1, *p*-value = 0.01004	
D-10	Rep1 dsGFP versus dsPara	Chi2 = 0.0050415, df = 1, *p*-value = 0.9434	Chi2 = 5.24, df = 8, *p* = 0,731
	Rep2 dsGFP versus dsPara	Chi2 = 1.7747, df = 1, p-value = 0.1828	
	Rep3 dsGFP versus dsPara	Chi2 = 0, df = 1, *p*-value = 1	
	Rep4 dsGFP versus dsPara	Chi2 = 0.64503, df = 1, *p*-value = 0.4219	

Summary data for all experimental replicates testing the effect of para gene silencing compared to control treatment with dsGFP on mosquito mortality rate post-deltamethrin exposure. Individual *p*-values were calculated per replicate by Chi2 statistical comparison between dsPara and dsGFP control conditions. If the replicates test were consistent (in the same phenotypic direction, see in Methods), then the individual *p*-values were combined by Fisher's method. The statistical significant combined *p*-values are in bold.

### Normal para function limits *Anopheles* susceptibility to *Plasmodium* infection

**Figure 2:**
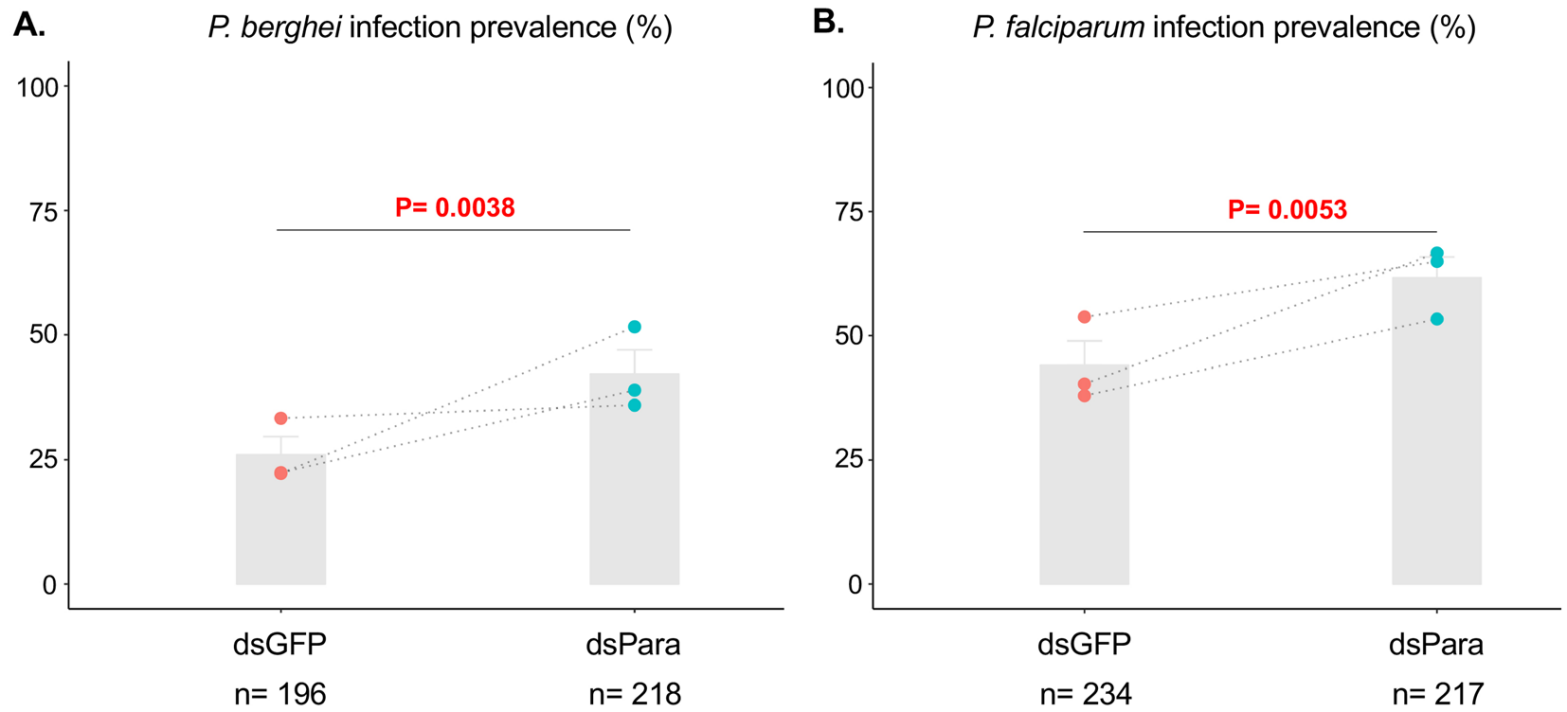
Para normal activity limits *Plasmodium* infection prevalence. Panel (**A**) and panel (**B**) show results of infection prevalence (proportion of infected mosquitoes) in *An. coluzzii* infected with *P. berghei* and *P. falciparum*, respectively. Grey color shows proportion of infected mosquitoes. Infection prevalence between dsPara and dsGFP was measured at d-8 post-infection and showed statistically significant increase in dsPara as compared to dsGFP control, for both *Plasmodium* species. Combined *p*-value (Fisher method) from the 3 independent biological replicates was indicated in each graph represented in both A and B panels. n = Total number of dissected mosquitoes. Values obtained in every biological replicate are depicted by dots and dotted lines.

We then queried the role of the VGSC on *Anopheles* vector competence for *Plasmodium* infection. *An. coluzzii* females were injected with dsPara or dsGFP for the control group and infected with the rodent malaria parasite *P. berghei* HSP70_GFP. *P. berghei* infection prevalence (proportion of infected mosquitoes) was significantly higher in the dsPara-treated mosquitoes as compared to the dsGFP controls (Chi2 = 19.252; *P*-value = 0.0038) (Fig. 2A). The same experiments performed with the human malaria parasite *P. falciparum* also resulted in an increase of infection prevalence at 8-d post-infection (Chi2 = 18.383; *P*-value = 0.0053) (Fig. 2B). There was no significant effect of dsPara treatment on infection intensity (the number of parasites in infected mosquitoes) for either *P. berghei* and *P. falciparum* (Fig.S2).

### Para activity limiting *Plasmodium *infection in *An. coluzzii* is microbiota dependent

We showed above that normal para levels are required for wild-type sensitivity to deltamethrin, as well as resistance to *Plasmodium* infection, because depletion of para caused decreased mortality due to insecticide and increased infection prevalence, respectively. To understand the mechanisms underlying these functions, we first determined the transcriptional effects of para depletion on mosquitoes, under the same conditions that displayed the functional effects. Transcriptomic analysis using RNAseq analysis of dsPara and dsGFP-treated mosquitoes, at 4 days post-dsRNA injection, indicated that only three genes were significantly differentially regulated between the two conditions (up: 20S proteasome subunit beta 5, NADH dehydrogenase subunit 2, down: annexin B10B; Sup data Table1, Sup Fig.S3). This result indicates that para activity displays a very small transcriptional footprint. This is particularly notable given the influence of para function limiting *Plasmodium* infection. Many immune genes are known to act against *Plasmodium* in a transcription-dependent fashion, but a transcriptional effect of para function on immune genes was not detected, indicating the absence of a direct link between para activity and immune gene expression. Thus, neither the effect of para on insecticide mortality nor on *Plasmodium* infection appear to be based strongly on transcriptional regulation and are likely have other explanations.

**Figure 3:**
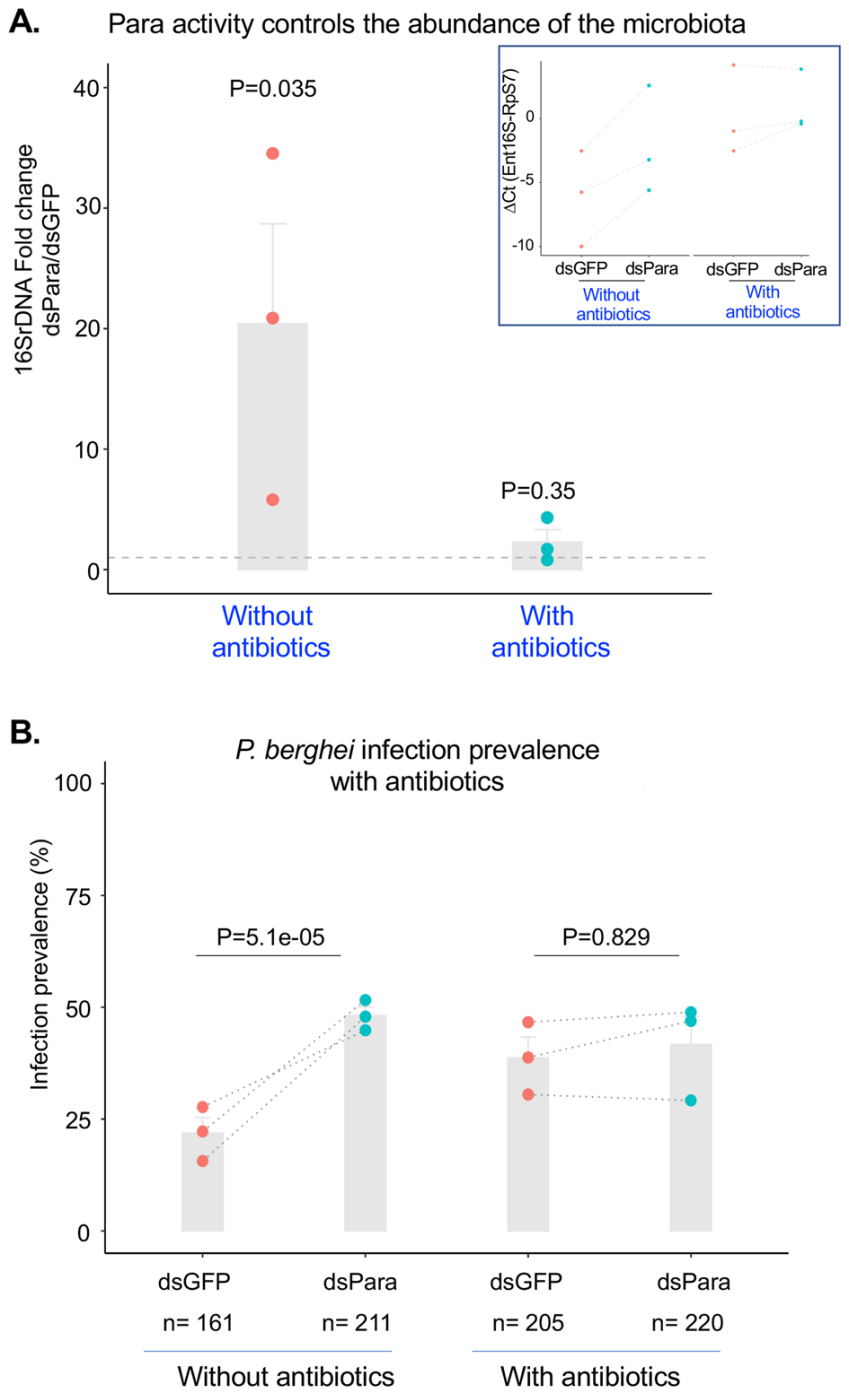
Para activity limiting *Plasmodium* infection is microbiota dependent. (**A**) 16srDNA relative fold change was measured by qPCR in samples collected from antibiotic treated and non-treated backgrounds. In the non-treated background, Para silencing leads to increased level of the total abundance of *An. coluzzii* bacteria as compared to the dsGFP control group (dotted line), whereas in antibiotic treated background this effect is abolished. The ratio of the normalized 16srDNA in dsPara/dsGFP was calculated using triplicates. *: Statistical *p*-value related to the deltaCt distribution between dsPara and dsGFP on 3 independent biological replicates are mentioned in the graph. (**B**) Infection prevalence between dsPara and dsGFP in antibiotic (ATB)-treated background was measured at d-8post infection and showed no statistical difference between the two conditions dsPara and dsGFP. Combined *p*-value (Fisher method) from the 3 independent biological replicates was obtained for the infection prevalence. n = Total number of dissected mosquitoes. Values obtained in every biological replicate are depicted by dots and dotted lines.

Bidirectional communication between gut bacteria and the brain, also referred as “gut-microbiota-brain axis” has been described to play a crucial role in the homeostasis of the gastrointestinal and microbial system of animals^43^. In addition, in mammals, VGSCs are expressed in primary neurons innervating visceral organs^44^. The enteric bacterial flora of mosquitoes is known to influence *Plasmodium* infection outcome^45–48^, and the abundance of Enterobacteriaceae is positively correlated with *Anopheles* vector competence for *Plasmodium*^45^. Therefore, we tested whether para depletion could alter the bacterial abundance and composition in the mosquito midgut. The enteric bacterial load was compared between dsGFP and dsPara backgrounds via quantification of 16S ribosomal RNA (16S rRNA) by qPCR. We observed that silencing of para led to increased overall bacterial abundance in the midgut, and, although this was not statistically significant, it specifically caused elevated abundance of taxa in the Enterobacteriacae (Fig. 3A, Fig S4).

We then queried the potential role of dsPara-dependent dysbiosis on *Plasmodium* infection outcome by treating mosquitoes with antibiotics. Para was depleted in *An. coluzzii* mosquitoes treated with antibiotic after adult emergence, and 4-d post-injection dsGFP and dsPara were infected with *P. berghei*. We found that antibiotic treatment reversed the effect of *para* depletion on *Anopheles* vector competence on both the enteric bacteria microbiota (Fig. 3A) and on *P. berghei* infection prevalence (Fig. 3B), as compared to the antibiotic-treated dsGFP controls (Fig. 3B). Antibiotic treatment did not affect *P. berghei* infection intensity (oocyst load) as previously observed in the absence of the treatment (Fig. S5). This result indicates that para activity controlling *Plasmodium* infection is microbiota dependent.

## Discussion

Here, we show that normal activity of the VGSC para is required for *An. coluzzii* sensitivity to killing by a pyrethroid insecticide, as well as to limit *Plasmodium* infection levels. Para activity appears to exert the influence on *Plasmodium* infection by altering the abundance and composition of the enteric bacterial microbiota. In mammals, excitation and contraction of gastrointestinal system muscles are regulated by membrane depolarization mediated by receptor channels^49^. Microbiota and gut motility are associated, and gastrointestinal motility can affect the microbiota in terms of amount, location and diversity^2,50–52^. In *Aedes* mosquitoes, the crop, a sugar-reach meal storage tissue, transfer the sugar into the digestive tract for digestion via peristaltic contractions, which are controlled by stretch-activated channels^53–55^. Stretch-activated channels include VGSC, therefore, we could hypothesize that Para participates in the contraction of the crop, which regulate carbohydrate resource distribution used for the mosquito itself, but these resources are also probably used by the enteric bacteria as an energy resource. As VGSCs participate in muscle contraction, the simplest explanation for our results, including the absence of direct transcriptional effect by para, is that para activity influences the abundance and composition of the enteric bacterial community by acting directly on the contractile motility of the *Anopheles* digestive tube or on the contractibility of the crop. 16S rRNA gene pyrosequencing of the microbiome between dsPara and dsGFP treated mosquitoes would help identifying bacteria taxa controlled by the VGSC activity, and the ones that are linked with the *Plasmodium* phenotype outcome. For example, higher titter of *Enterobacteriaceae* were significantly correlated with *P. falciparum* infection^45^.

Binding of the pyrethroid deltamethrin to the VGSC channel fixes the receptor in its active conformation, which leads to constitutive depolarization of the cell membrane and insect death. In *Drosophila*, para transgenic deletion led to paralysis and decreased lifespan^21,22^. However, in *Anopheles* a lethality effect was not detected after transient partial depletion of para transcript by RNAi, likely because residual para protein maintained partial physiological function of the VGSC. We also showed that reduced activity of para by RNAi-mediated silencing increased *Plasmodium* infection prevalence. Therefore, it could be possible that augmentation of para activity could decrease *Anopheles* competence for *Plasmodium* infection. Consistent with their implication in immune-related functions in mammals, VGSC blockers has been proposed as immune modulators for clinical applications^56^. Therefore, designing an insecticide structural analog or an allosteric ligand that could act as a selective para agonist, but without blocking the receptor channels in a lethal conformation, could potentially generate a tool to control malaria infection of mosquitoes, and therefore limit transmission.

In *Anopheles*, some observations have associated the occurrence of *kdr* mutations with increased *P. falciparum* infection^27,29,31^, while no relationship was observed in another study. If we assume, that kdr mutations may alter the function and activity of para, and the mosquito vector competence, the presence/absence of correlation found in these studies could be attributed to the composition of the enteric bacteria flora between the mosquitoes from these distinct studies. Previously we identified a genetic locus, named Pfin6, associated with mosquito infection in nature by the human malaria parasite *P. falciparum*^31^. In this locus, the haplotype carrying the insecticide resistant kdr allele of para was linked to increased parasite infection prevalence. However, we do not know whether allelic variations in para could alter its physiological function, and thus its role in *Plasmodium* development, which may explain these distinct results found in the association studies mentioned above and performed from different mosquito genetic backgrounds. In the current study, depletion of para phenocopied the kdr effect by decreasing mortality after deltamethrin exposure and increases *Plasmodium* infection prevalence. The 1014F mutation underlying the kdr allele was introduced using CRISPR/Cas9 genome editing into an insecticide susceptible *Anopheles* colony^57^, but the influence on *Plasmodium* infection was not tested, which would be interesting. It could also be informative to test the effect of RNAi-mediated para depletion, as done here, but in a mosquito colony fixed for different kdr and related mutations, and test their susceptibility to *Plasmodium* and effect on enteric bacterial flora.

Transcriptome analysis indicated that para activity influences transcript abundance of only three other genes. Annexin B10B, which was downregulated after para silencing, belongs to a family that can bind to *Plasmodium* ookinetes, and annexin B10B expression is significantly decreased 48 h after a bloodmeal^58,59^, but it is not known whether annexin binding influences *Plasmodium* infection. The two genes induced by para depletion code for the subunit beta 5 of the 20S proteasome, which is responsible for protein degradation, and NADH dehydrogenase subunit 2. Functional analyses of these genes by RNAi silencing and *Plasmodium* infection challenge will be required to clarify their potential role in *Plasmodium* infection.

Finally, in the context of the brain-gut-microbiome axis well studied in mammals, identifying new mosquito receptors from the insect nervous system known to control cell excitability such as neurons and muscles, could be tested for their effects on the mosquito microbiome. For example, testing whether others receptor channels or G-protein coupled receptors known to control brain activity could modulate the composition of the mosquito microbiome, and indirectly *Anopheles* vector competence, is completely unexplored. Functional study through RNAi combined with pharmacological tool using known agonists or antagonists of these receptors could be used to explore this research field and represent molecule candidate for controlling malaria transmission.

### Supplementary Information

Supplementary Information 1.

Supplementary Information 2.

Supplementary Information 3.

Supplementary Information 4.

Supplementary Information 5.

Supplementary Information 6.

Supplementary Information 7.

## Data Availability

Sequence data related to analysis are available under the ArrayExpress accession E-MTAB-12896. URL link: https://www.ebi.ac.uk/biostudies/arrayexpress/studies/E-MTAB-12896?key=8617f493-4d74-4aff-987f-66470012a884.
