# Supplementary figures and images for: The voltage-gated sodium channel, para, limits Anopheles coluzzii vector competence in a microbiota dependent manner
Source: Sci Rep. 2023 Sep 4;13:14572. doi: 10.1038/s41598-023-40432-x (PMC10477260; doi:10.1038/s41598-023-40432-x)

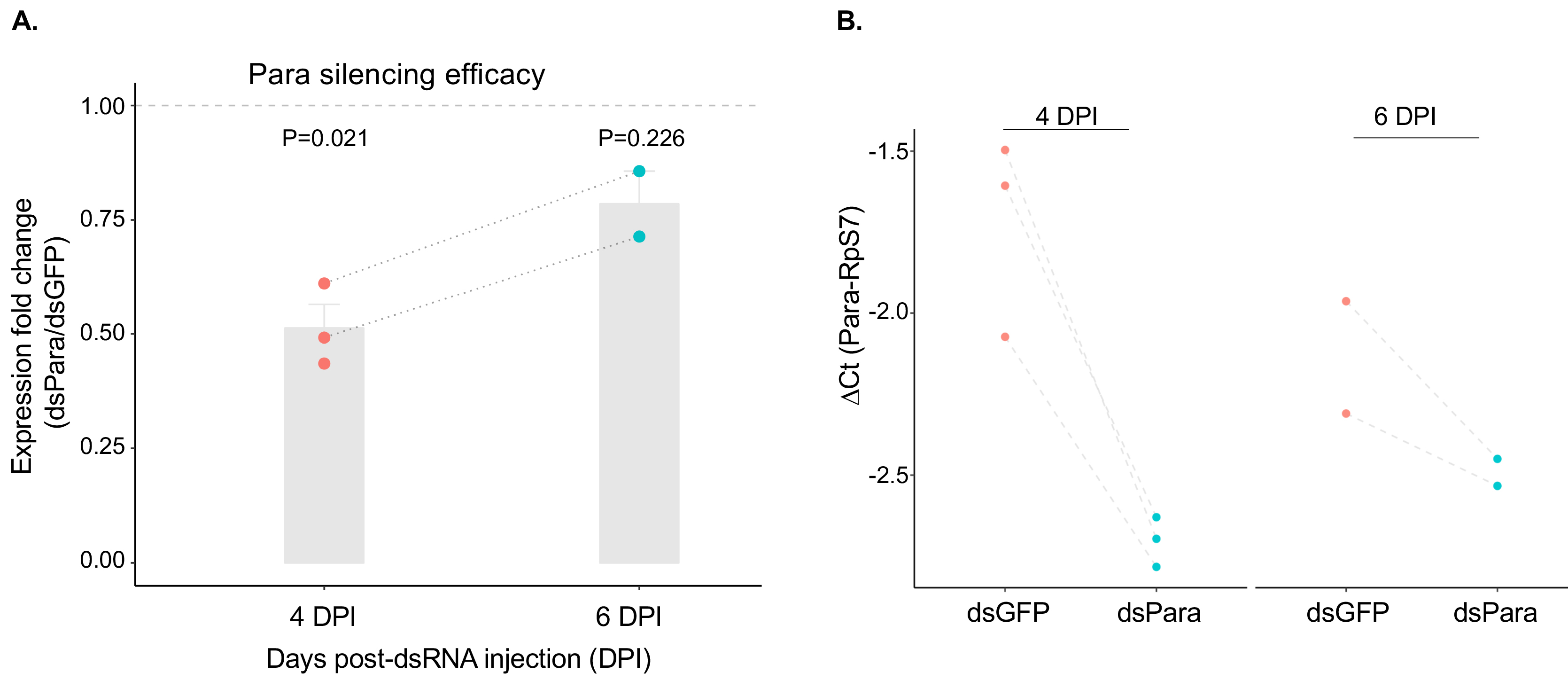

Supplement: Supplementary file 1 — Supplementary Information 1. [file 41598_2023_40432_MOESM1_ESM.jpg]

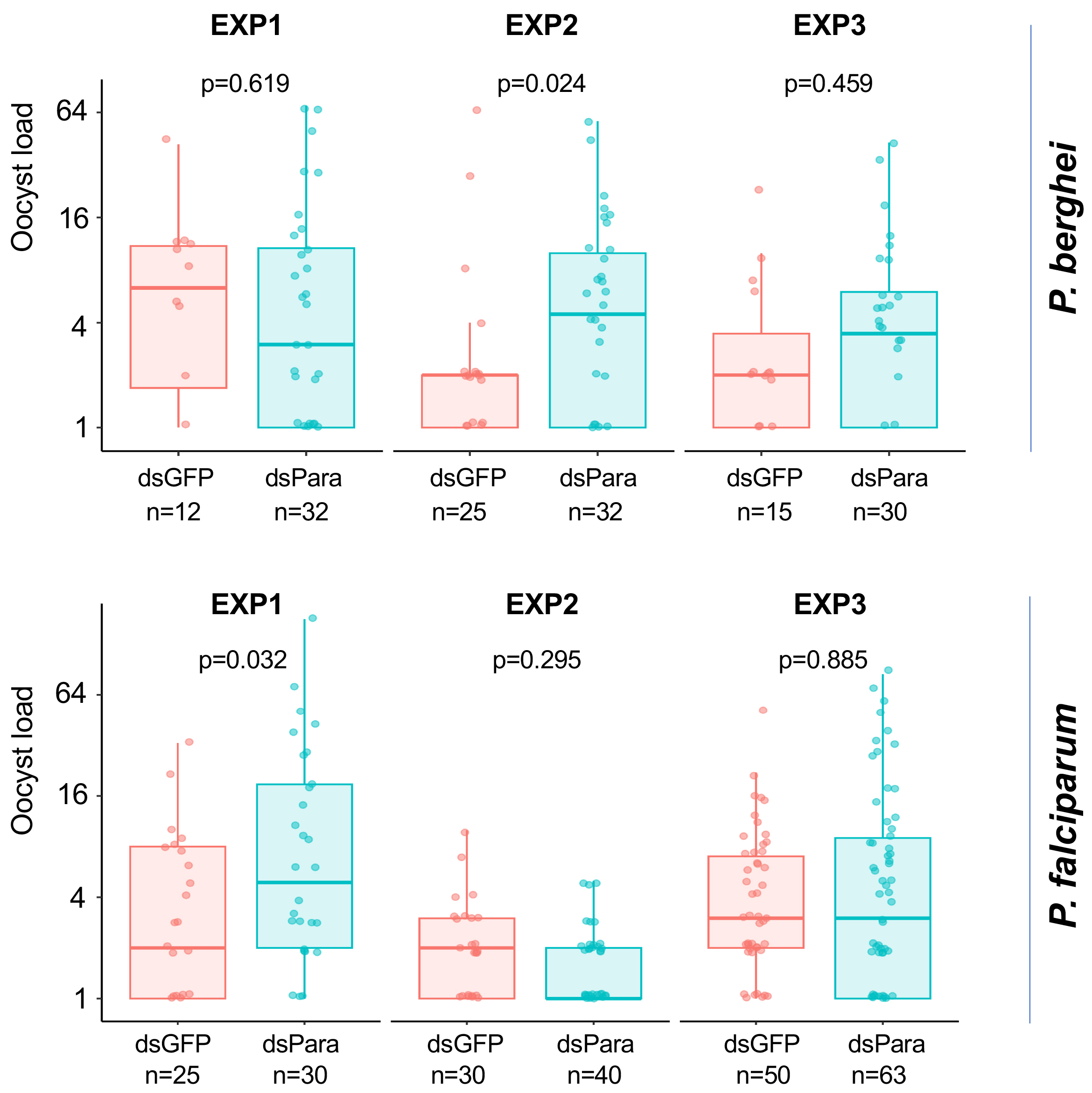

Supplement: Supplementary file 2 — Supplementary Information 2. [file 41598_2023_40432_MOESM2_ESM.jpg]

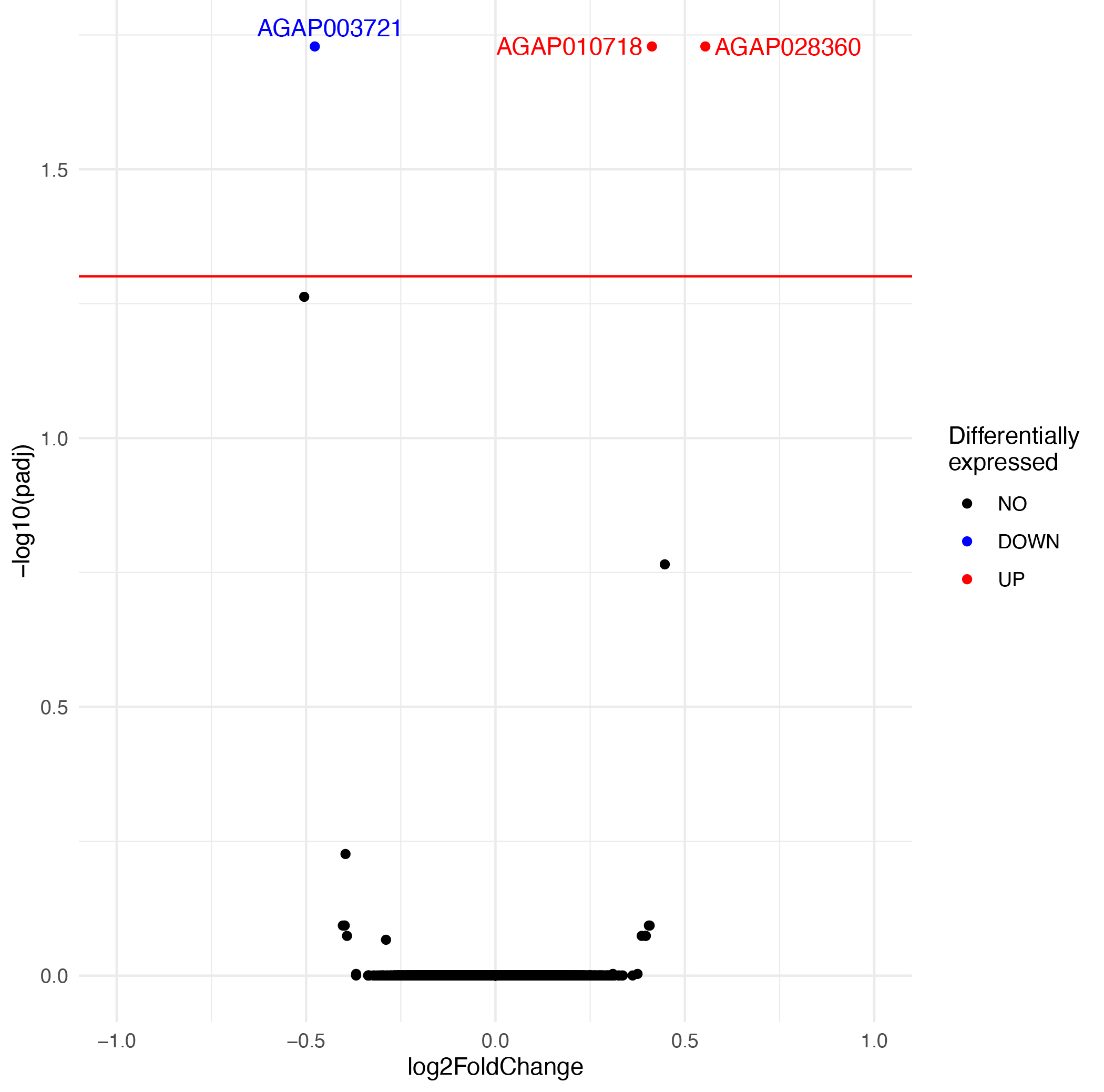

Supplement: Supplementary file 3 — Supplementary Information 3. [file 41598_2023_40432_MOESM3_ESM.jpg]

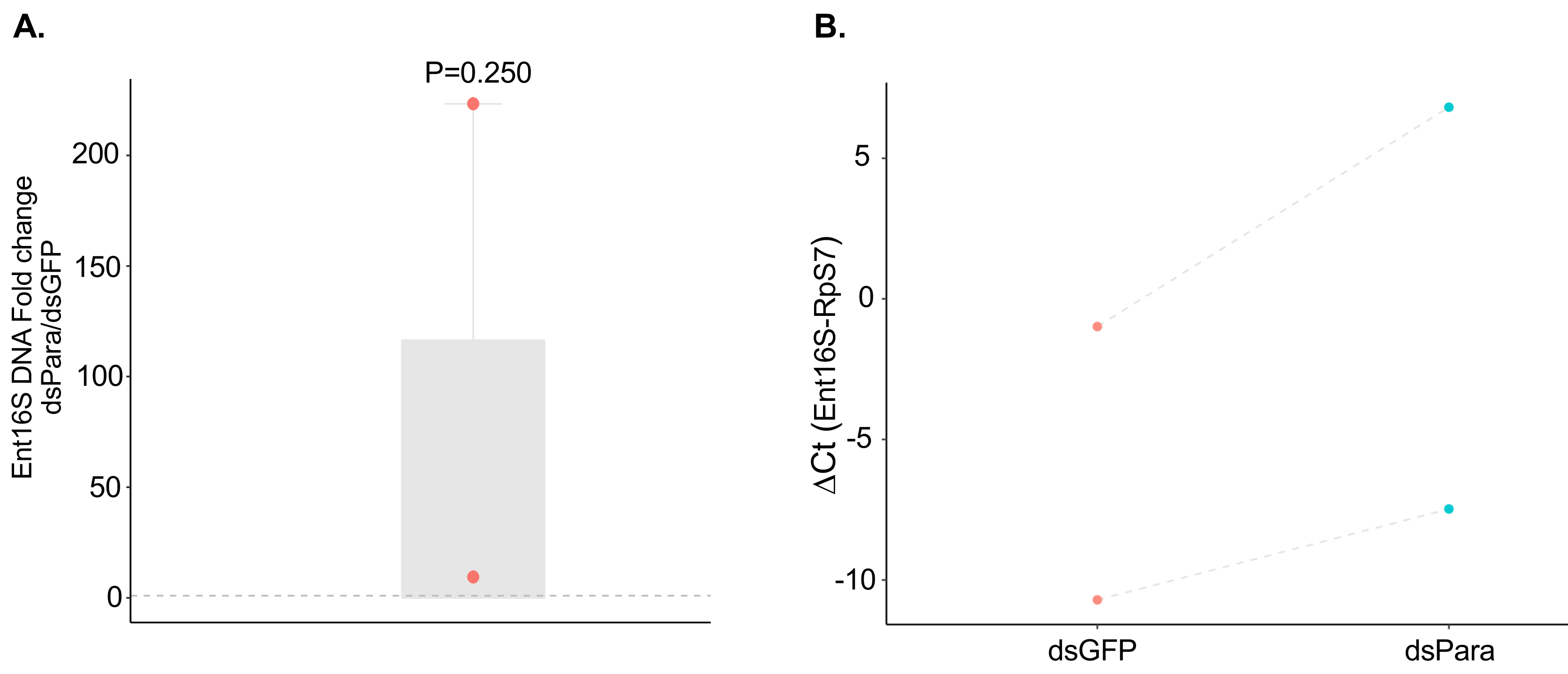

Supplement: Supplementary file 4 — Supplementary Information 4. [file 41598_2023_40432_MOESM4_ESM.jpg]

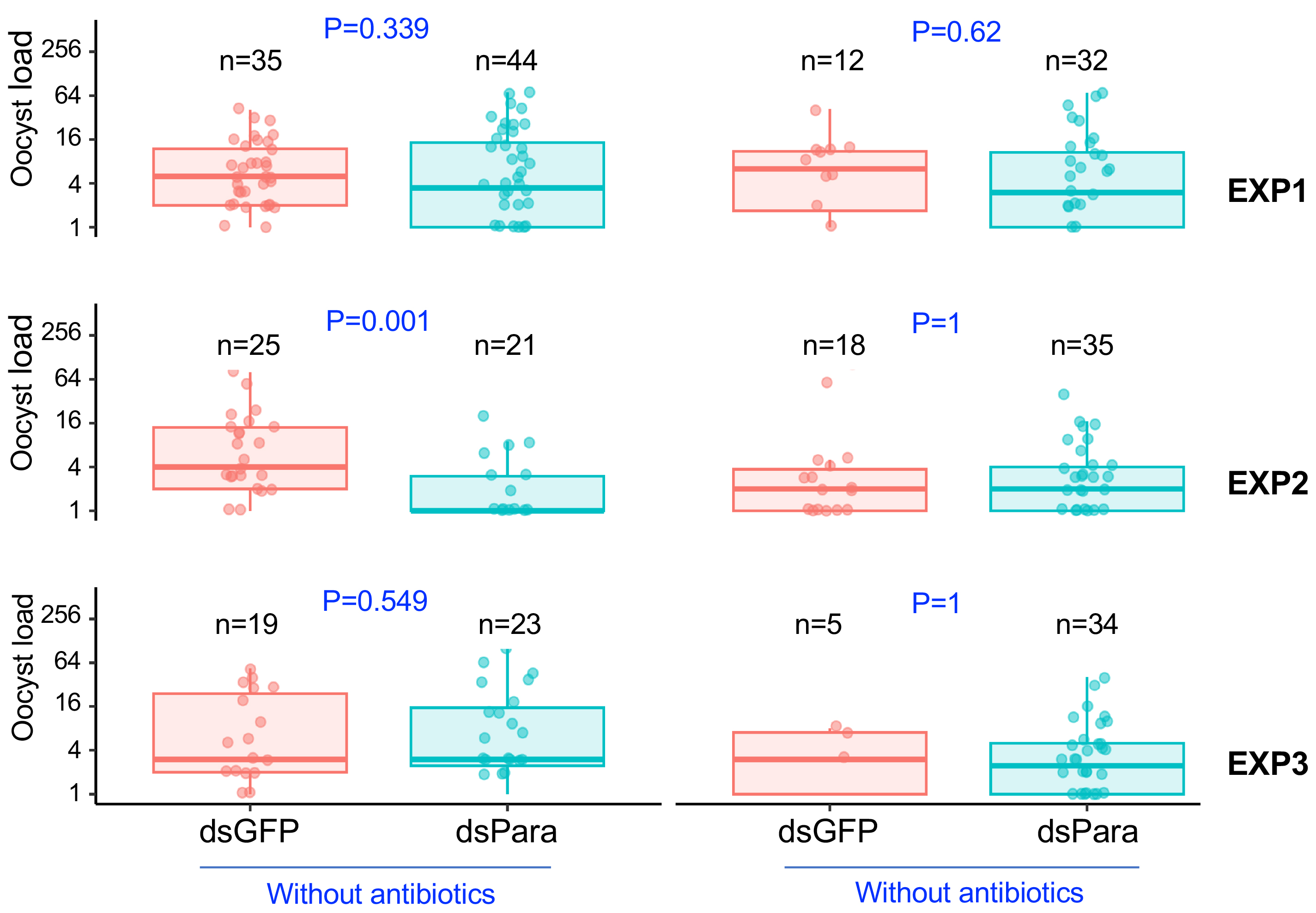

Supplement: Supplementary file 5 — Supplementary Information 5. [file 41598_2023_40432_MOESM5_ESM.jpg]
