# Supplementary material for: The voltage-gated sodium channel, para, limits Anopheles coluzzii vector competence in a microbiota dependent manner
Source: Sci Rep. 2023 Sep 4;13:14572. doi: 10.1038/s41598-023-40432-x (PMC10477260; doi:10.1038/s41598-023-40432-x)
Supplement: Supplementary file 6 — Supplementary Information 6. [file 41598_2023_40432_MOESM6_ESM.docx]

**The voltage-gated sodium channel, para, limits *Anopheles coluzzii* vector competence in a microbiota dependent manner**

**Legends for supplementary information**

**Figure S1:** **RNAi efficiency of dsPara:** A. The graph shows relative fold change of para expression at d-4 and d-6 post-injection (DPI) between dsRNA (dsPara) that target all the different transcript variants, and the control dsRNA (dsGFP) (dotted line). RpS7 was used as an internal reference. For the detection of para transcripts, qPCR primers that amplify a genomic region shared by all the 13 transcript variants were used. Error bars show median absolute deviation computed by permutation from 3 experiments at d-4 and 2 experiments at d-6. Statistical p-value related to the deltaCt distribution between “dsPara” and “dsGFP” are mentioned for each DPI. B. The graph shows variation of ∆Ct between Para and RpS7, the internal reference in both dsRNA treated backgrounds. Values obtained in every biological replicate are depicted by dots and dotted lines.

**Figure S2:** ***Plasmodium* infection intensity**: Infection intensity between dsPara and dsGFP backgrounds was measured at d-8 post infection. Three independent replicates were performed for both *P. berghei* and *P. falciparum*. Wilcoxon p-values are mentioned for each replicate experiment (EXP). The results showed no consistency for the phenotype direction across the three replicates, therefore the p-value were not combined according to the Fisher method. n = Total number of dissected mosquitoes that carry at least one oocyst parasite in each EXP. Y-axis is log2-scaled. Values obtained in every biological replicate are depicted by dots.

**Figure S3: Para activity displays a very small transcriptional footprint.** The figure represents a Volcano plot illustrating transcriptomic analysis comparing differential gene expression between dsPara and dsGFP mosquitoes. Each black dot represents the differential expression of a gene between dsPara and dsGFP and is illustrated as the logarithm (log) of adjusted p-value (Padj) in function of magnitude of expression fold change (log2 Foldchange). Red dots represent genes statistically significantly upregulated in dsPara as compared to dsGFP control. The blue dot represents the gene statistically significantly downregulated in dsPara as compared to dsGFP control.

**Figure S4: Normal para activity limits Enterobacteriaceae abundance increase. A.** Enterobacteriaceae DNA (Ent16S) fold change was measured by qPCR and show increased level of the total abundance of *A. coluzzii* bacteria in dsPara as compared to the dsGFP control group. The ratio of the normalized Ent16S DNA in dsPara/dsGFP was calculated using triplicates. p-value related to the deltaCt distribution between dsPara and dsGFP was performed using a Student t-test. B. The graph shows variation of ∆Ct between Ent16S and RpS7, the internal reference in both dsRNA treated backgrounds. Values obtained in every biological replicate are depicted by dots and dotted lines.

**Figure S5:** ***Plasmodium* *berghei* infection intensity with and without antibiotics**: Infection intensity between dsPara and dsGFP backgrounds, without and with antibiotic treatment, was measured at d-8 post infection. Three independent replicates were performed for *P. berghei* infection. Wilcoxon p-values are mentioned for each replicate experiment (EXP) and for each treatment (with and without atibiotics). The results showed no consistency for the phenotype direction across the three replicates, therefore the p-value were not combined according to the Fisher method. n = Total number of dissected mosquitoes that carry at least one oocyst parasite in each EXP. Y-axis is log2-scaled. Values obtained in every biological replicate are depicted by dots.

**Supplementary table 1: Transcriptome analysis comparing dsPara and dsGFP mosquitoes**

For each gene, differential expressions between dsPara and dsGFP backgrounds are expressed in Log2 fold change, p-values and adjusted p-values (padj) to highlight statistical difference. The count values are noted for each gene and for each three biological replicates: GFP_1 = samples from mosquitoes treated with dsGFP and collected at 4 days pos-injection for the first biological replicate; Para_1= samples from mosquitoes treated with dsPara and collected at 4 days pos-injection for the first biological replicate.
